# A microfluidic-based PDAC organoid system reveals the impact of hypoxia in response to treatment

**DOI:** 10.1038/s41420-023-01334-z

**Published:** 2023-01-21

**Authors:** Marlene Geyer, Daniel Schreyer, Lisa-Marie Gaul, Susanne Pfeffer, Christian Pilarsky, Karla Queiroz

**Affiliations:** 1 MIMETAS BV, De Limes 7, 2342DH Oegstgeest, The Netherlands; 2 School of Cancer Sciences, University of Glasgow, Garscube Estate, Switchback Road, Bearsden, G61 1BD Glasgow, United Kingdom; 3 Universitätsklinikum Erlangen, Schwabachanlage 12, 91054 Erlangen, Germany

**Keywords:** Pancreatic cancer, Cell signalling

## Abstract

Pancreatic Ductal Adenocarcinoma (PDAC) is estimated to become the second leading cause of cancer-related deaths by 2030 with mortality rates of up to 93%. Standard of care chemotherapeutic treatment only prolongs the survival of patients for a short timeframe. Therefore, it is important to understand events driving treatment failure in PDAC as well as identify potential more effective treatment opportunities. PDAC is characterized by a high-density stroma, high interstitial pressure and very low oxygen tension. The aim of this study was to establish a PDAC platform that supported the understanding of treatment response of PDAC organoids in mono-, and co-culture with pancreatic stellate cells (PSCs) under hypoxic and normoxic conditions. Cultures were exposed to Gemcitabine in combination with molecules targeting relevant molecular programs that could explain treatment specific responses under different oxygen pressure conditions. Two groups of treatment responses were identified, showing either a better effect in monoculture or co-culture. Moreover, treatment response also differed between normoxia and hypoxia. Modulation of response to Gemcitabine was also observed in presence of a Hypoxia-inducible factor (HIF) prolyl hydroxylase (PHD) inhibitor and HIF inhibitors. Altogether this highlights the importance of adjusting experimental conditions to include relevant oxygen levels in drug response studies in PDAC.

## Introduction

PDAC is characterized by high-density stroma that makes up to 90% of the tumor volume and consists of cancer-associated fibroblasts (CAFs), adipocytes, immune, endothelial, nerve and pancreatic stellate cells (PSCs) as well as extracellular matrix components (ECM) such as collagen and hyaluronic acid [1]. All these cells interact with the tumor to modify its behavior. Apart from the dense stroma, PDAC is also poorly vascularized and presents abnormal leaky blood vessels. PSCs are the most studied stromal component in PDAC, and these are named after their star-like shape. In normal pancreas, these cells are quiescent and store vitamin A, whereas in PDAC these get activated and change into a myofibroblast like cell. Activated PSCs express ECM components like collagen I, alpha smooth muscle actin (ɑ-SMA), fibronectin and transforming growth factor (TGF)-β, together this supports proliferation, inflammation and induces desmoplasia [2]. PSCs have been shown to increase collagen production in response to hypoxia contributing to the formation of a fibrotic stroma and preventing immune cell recruitment [3]. Several studies have suggested that the stroma might represent a defense mechanism at first, however it contributes later to cancer progression where excessive stroma content causes pressure on vasculature and decreases the oxygen content and nutrient flow [4].

Healthy pancreatic tissue has an oxygen pressure of 30–50 mmHg, which is decreased to 2.5 mmHg in solid tumors. PDAC is considered severely hypoxic, with 0.7% oxygen content, however hypoxic sites are heterogeneously distributed throughout the tumor tissue [5, 6]. Therefore, it is important to adjust experimental in vitro conditions to simulate relevant PDAC oxygen levels. Chang et al. found a correlation between higher hypoxia levels with increased growth and proliferation. In addition, hypoxia is associated with epithelial-to-mesenchymal transition (EMT) and consequent increased metastatic potential [7]. Hypoxia induces a metabolic switch from oxidative phosphorylation to lactate production contributing to acidification of the tumor microenvironment. HIF-1α is a hypoxia marker and consists of three isoforms, where each form can dimerize with constitutively expressed HIF-1β and then binds to hypoxia related genes. [8]. Onishi et al. showed increased Hedgehog (Hh) signaling activation which seems to contribute to tumor invasiveness. Hypoxia also promotes the expression of various genes such as matrix metalloproteinases (MMPs), vascular endothelial growth factor (VEGF), N-cadherin through nuclear factor kappa B (NF-KB) pathway as well as transcription factors Snail, Twist and Slug [9–11]. Also, the activated Wnt, Notch and PI3K/Akt/mTOR pathways play a role in inducing chemoresistance upon stemness activation [12]. Moreover, the expression of Fascin and LASP-1 (LIM And SRC Homology 3 Domain (SH3 Protein) 1) as well as mitogen-activated protein kinase (MAPK) pathway are activated [13]. Hypoxia also upregulates glucose transporters GLUT1/3 in PSCs [14] as well as increases migratory ability, collagen I and VEGF production. Even conditioned medium of PSCs grown in hypoxia leads to changes such as cell proliferation, angiogenesis, and migration in experiments [15]. Together these studies support the notion that hypoxic tumors have an increased metastatic potential, chemoresistance and a consequent poor prognosis [16]. Desmoplasia, high interstitial pressure, and resulting hypoxia-driven molecular programs are relevant players in rendering PDAC tumors untreatable. Taking this into account, our study envisioned to establish PDAC organoid-based models that included stromal cells as well as oxygen tension that simulate that of the tumor core. We here used a 3D microfluidic platform to establish models composed by PDAC and PDAC-PSCs co-cultures, which were cultured in normoxic and hypoxic conditions. Phenotypic and transcriptional changes associated to hypoxia were observed. In addition, treatment responses were dependent on oxygen tension, indicating that hypoxic conditions should be adjusted and could support a better understanding of treatment failure as well as opportunities to effectively treat PDAC.

## Results

### Development and characterization of a PDAC-PSC co-culture model on a microfluidic platform

**Fig. 1:**
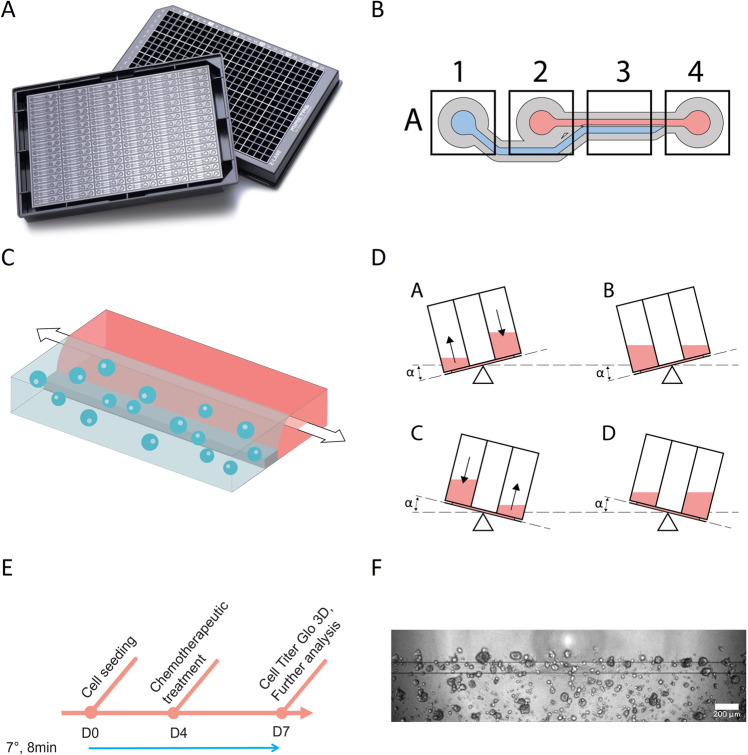
Development of PDAC-on-a-Chip models. **A** Image of the OrganoPlate® 2-lane from MIMETAS and (**B**) a zoomed in schematic overview of one chip. The gel inlet (A1) is connected to the gel channel (blue). The perfusion channel (red) connects the perfusion inlet (A2) with the perfusion outlet (A4). **C** Schematic representation of a chip filled with PDAC organoids and PSCs. The cells are mixed with extracellular matrix, and these are seeded in the gel channel upon pipetting into the gel inlet and subsequently distributed along the gel channel due to capillary forces. The channels are separated by Phaseguides, capillary pressure barriers, which keep the channels separate from each other and allows the stratified loading of culture components. After gelation, cell culture medium was added to the medium inlet and outlet. During all experiments, when the plates were in the incubator (37 °C), these were kept on an (**D**) interval rocker at an inclination of 7° and an 8-min interval. The interval rocker ensured perfusion of the cultures. To enhance optical clarity, 50 μl of Hanks Balanced Salt Solution (HBSS) (Sigma, 55037 C) was dispensed into the observation windows of the OrganoPlate® 2-lane. **E** Timeline of the experiment: On day 0 PDAC organoids and PSCs were seeded in Matrigel suspension, cells were allowed to expand until day 4, when these were subjected to chemotherapeutic treatment for 72 h. Cell survival was analyzed using Cell Titer Glo 3D Viability assay. **F** Representative Phase Contrast (PC) image of a PDAC organoid monoculture in an OrganoPlate 2-Lane. 4x acquisition, Scale bar: 200 um. PC Images were acquired on the ImageXPress Micro XLS Widefield High-Content Analysis System® (Molecular Devices, US).

Complex tumor models are essential for effective drug discovery and development. This is particularly relevant for tumors which lack effective treatments such as PDAC. For this study, PDAC organoids were 3D grown in a microfluidic platform under flow conditions ensuring nutrient distribution and waste removal. In addition, PSCs were included to mimic the vital role of the stroma in PDAC, and its role in preventing effective response to treatment. PDAC organoids were loaded into the gel channel (compartment A1) of the OrganoPlate® 2-Lane (Fig. 1A, B) either in monoculture or in co-culture with PSCs in a ratio of 2:1 (PDAC organoids: PSCs) in GFR-Matrigel and enabled to generate a 3D culture. Medium was distributed into the top channel by adding it to the medium inlet and outlet (Fig. 1B: A2 and A4). The 2:1 seeding ratio was chosen due to the slow growing nature of the organoids and the fast-growing PSCs. It is estimated that at the beginning of the drug exposure studies the stromal component makes up to 80% of the culture. These cultures were grown in hCPLT-1 medium (Table S1) during the 7-day experiment. The cells seeded in the microfluidic chip were subjected to bidirectional perfusion flow (Fig. 1C, D). Moreover, to properly mimic the PDAC tumor microenvironment cultures were maintained for 4 days in either hypoxic (1% O_2_) or normoxic (20% O_2_) conditions until drug screenings were performed for another 72 h (Fig. 1E).

**Fig. 2:**
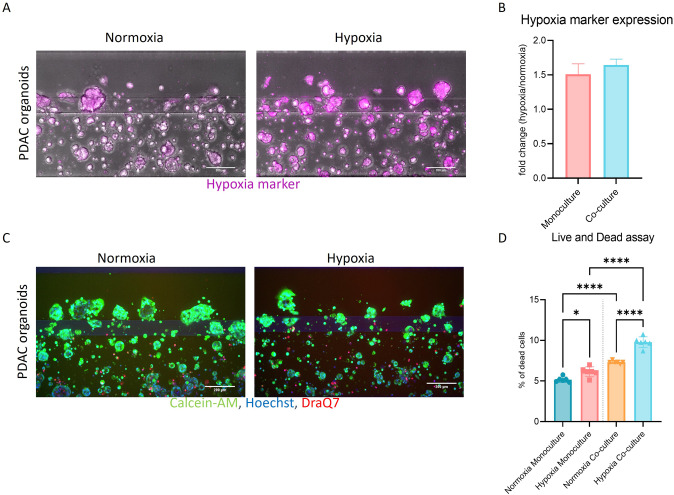
Assessment of hypoxia and cell viability in PDAC organoids cultures and PDAC organoids-PSCs co-cultures. Cells were grown for 4 days in the OrganoPlate® 2-Lane until used for subsequent analyses. **A** shows representative images of a hypoxia marker staining, images were acquired by confocal microscopy. **B** Hypoxia probe fluorescence intensity was quantified and data normalized to the probe in normoxic conditions. Data is shown as fold change of normoxic conditions (*N* = 3, *n* = 3). **C** Live and Dead assay staining with Hoechst, DraQ7 and Calcein-AM was used to access viability of the cultures. **D** shows the percentage of dead cells compared to the total cell number in cultures (*N* = 3, *n* = 3). The data was compared with Ordinary one-way ANOVA and Tukey’s multiple comparison test and shown are mean and SD (****p ≤ 0.0001, ***P ≤ 0.001, **P ≤ 0.01, *P ≤ 0.05). Confocal images were acquired on the ImageXpress Micro Confocal (Molecular Devices, US).

Cells cultured under acute hypoxia and normoxia were utilized to investigate the phenotypic and transcriptional changes. 3D co-cultures were cultivated for 7 days, subsequently fixed and immunostained. Representative confocal images are included in Fig. S1B and revealed the expression of Cytokeratin 19 (CK19) in PDAC organoids and of Vimentin and ɑ-SMA in PSCs, indicating their activation (Table S2). Phase contrast images show that PSCs grew well over the 7-day time-course, although due to Matrigel embedding proliferation of these cells should be decreased in comparison to 2D cultures [17] (Fig. S1A). Hypoxia was confirmed by an Image-iT™ Red Hypoxia probe, fluorescence intensity quantification showed an increase of hypoxia of 1.5 and 1.8 times in mono- and co-cultures, respectively (Fig. 2A, B). Live and dead staining indicated well-growing cultures (Fig. 2C, D) in both normoxia and hypoxia. However, hypoxic cultures seemed to grow slower in both culture conditions and presented a slight increase in dead cells.

### Hypoxia-induced phenotypic and genotypic changes in PDAC organoids (co-)cultures

**Fig. 3:**
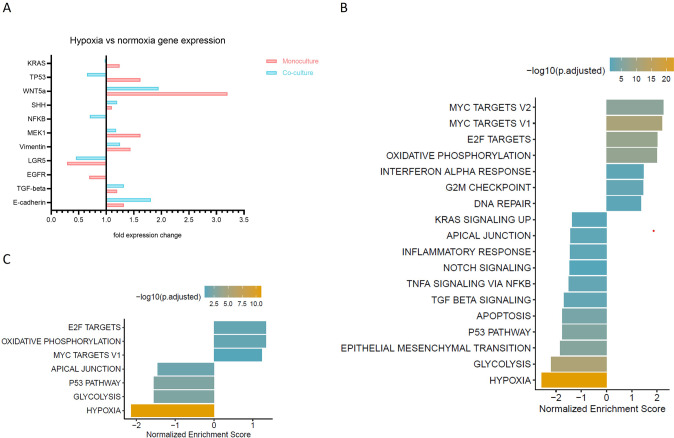
Hypoxia influences the transcriptional state of PDAC organoids (co-)cultures. Cells were grown under 1% O_2_ or 20% O_2_, for 4 days until harvested for RNA isolation, cDNA synthesis and qPCR. **A** Gene expression analysis of cells grown in hypoxia vs. normoxia. Fold expression changes in normoxia and hypoxia in monoculture and co-culture. The values were normalized to β-actin expression and to normoxic culture conditions to determine the expression differences in hypoxia. (*N* = 3, 24 chips were pooled for each sample) **B** RNA Sequencing data of PDAC organoids grown in normoxia and hypoxia. The data is depicted with the Hallmark database and shows pathways upregulated in normoxia (above 0) and pathways upregulated in hypoxia (below 0). The color indicates the -log10(p.adjusted) (*N* = 1, 48 chips were pooled for each sample). **C** RNA-Sequencing data of PDAC organoids in co-culture with PSCs grown in normoxia and hypoxia. The data is depicted with the Hallmark database and shows pathways upregulated in normoxia (above 0) and pathways upregulated in hypoxia (below 0). The color indicates the -log10 (p.adjusted). (*N* = 1, 48 chips were pooled for each sample). Pathways with *P* < 0.05 are depicted. The graph with all up-, and downregulated genes are depicted in Fig. S2.

To further evaluate the crucial genes and pathways affected by different oxygen tension conditions, gene expression analysis was performed with cells grown under normoxic and hypoxic conditions for 4 days. Subsequent qPCR analysis was employed to determine specific gene expression levels. Moreover, monoculture and co-culture samples were subject to RNA sequencing and differential gene expression established (Fig. 3).

Gene expression analysis revealed that KRAS, TP53, WNT5a, SHH, MEK, Vimentin, TGF-β and E-cadherin are upregulated in PDAC monocultures under hypoxia. Genes of epithelial origin such as KRAS, TP53, NFKB and LGR5 are reduced in co-culture under hypoxic conditions. This is likely a result of the decreased number of PDAC organoids in co-cultures at the end of the experiment, when co-cultures are composed mostly of PSCs. LGR5 was also downregulated under hypoxia. WNT5a, SHH, MEK, Vimentin, TGF-β and E-cadherin expression is increased in co-cultures under hypoxia (Fig. 3A). Cultivating cells in hypoxia resulted in a clear upregulation of pathways involved in KRAS signaling, inflammatory response, TGF-β signaling, apoptosis, P53, EMT, glycolysis and hypoxia in monoculture (Fig. 3B), confirming qPCR results. In co-culture conditions, similar pathways have been activated in response to hypoxia, confirming that also PSCs undergo similar cell transcriptional changes (Fig. 3C). In addition, pathways upregulated in normoxia, including oxidative phosphorylation, DNA repair and Myc-targets confirm the *normoxic* transcriptional state (Fig. 3B, C).

### (Chemo)therapeutic treatment response of PDAC organoids in monoculture and co-culture under diverse oxygen levels

Although Gemcitabine remains the standard-of-care agent in PDAC, treatment responses are rarely complete due to resistance and ineffectiveness of the drug and only around 4% of patients survive two years on the treatment alone [18]. A strategy to overcome Gemcitabine resistance is to combine it with other therapeutics that target specific PDAC dependent genes/pathways.

After the establishment of co-culture conditions, these were subjected to several combinatorial treatments, initiated four days after cell seeding Gemcitabine (1 µM) was combined with the following compounds (1 µM): Trametinib (MEK inhibitor), PD025901 (MEK inhibitor), MK-2206 (AKT inhibitor), SN38 (DNA topoisomerase inhibitor), Syrosingopine (MCT1/MCT4 dual blocker), Metformin (GPD2 inhibitor), and Erlotinib (EGFR inhibitor) (Table S3). Drug exposure was performed under normoxic and hypoxic conditions.

**Fig. 4:**
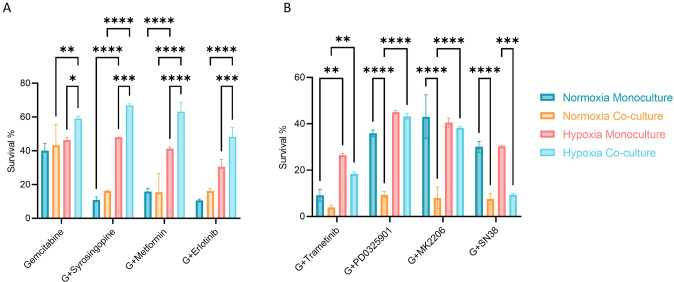
Chemotherapy treatment effect under different oxygen tension. PDAC organoids were grown in monoculture and co-culture with PSCs under normoxia and hypoxic conditions. The cells were grown for 4 days on the OrganoPlate® and subjected to several combinations of (chemo)therapeutics for 72 h, after which their survival was analyzed with Cell Titer Glo 3D viability assay. The graphs show the survival of the cultures normalized to a no-treatment control (medium only). Cells were subject to 1 µM Gemcitabine (G) alone and in combination with other compounds (shown in graphs **A** and **B**). Shown are mean + − SD and the data points represent individual chips (*N* = 3, *n* = 3). Statistical analysis shows results of a 2-way ANOVA with Tukey’s multiple comparisons (****p ≤ 0.0001, ***P ≤ 0.001, **P ≤ 0.01, *P ≤ 0.05).

Drugs were divided into two groups according to the type of response observed in mono- or co-cultures. Group A is composed by Gemcitabine and combinatorial treatment with Syrosingopine, Metformin and Erlotinib (Fig. 4). These compounds were more active in PDAC monocultures, suggesting a protective effect of the PSCs towards the PDAC organoids or less important influence of these pathways in co-culture conditions. Group B containing Trametinib, PD0325901, MK2206 and SN38 (Fig. 4), affected more co-culture survival compared to monocultures, suggesting that PSCs are more susceptible to the targeting of kinases such as MEK and AKT as well as DNA topoisomerase (Table S4).

**Fig. 5:**
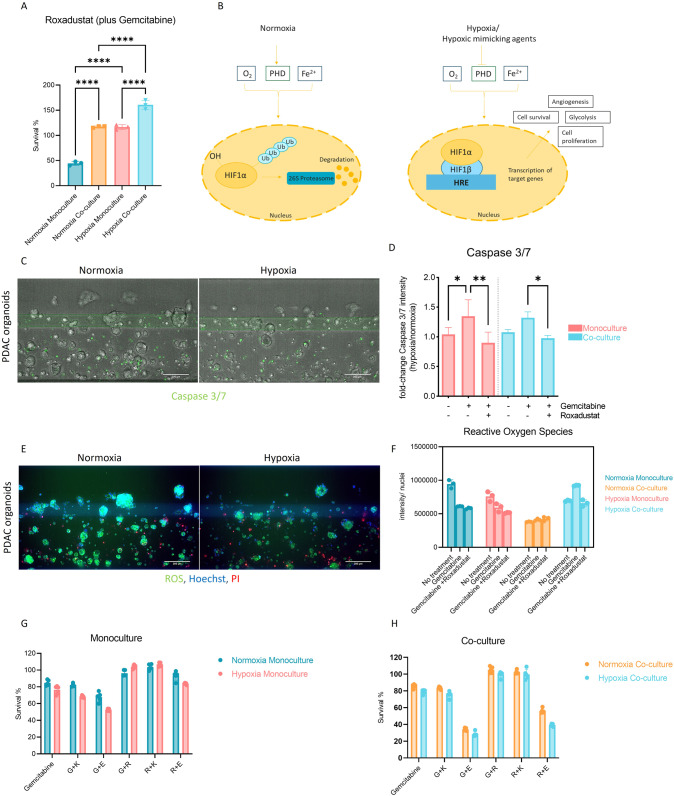
Effect of hypoxia and hypoxia-mimicking compounds on PDAC response to treatment. **A** PDAC organoids were grown in monoculture and co-culture with PSCs under normoxic and hypoxic conditions. The cells were grown for 4 days on the OrganoPlate® and subjected to 1 µM Gemcitabine and 1 µM Roxadustat for 72 h, before their survival was analyzed with Cell Titer Glo 3D viability assay. The graph shows the survival of the organoids normalized to a no-treatment control (=medium only). 0.1% DMSO was used as a vehicle control and showed no effect on the cells. The data was compared with Ordinary one-way ANOVA and Tukey’s multiple comparison test and shown are mean and SD (****p ≤ 0.0001, ***P ≤ 0.001, **P ≤ 0.01, *P ≤ 0.05). **B** Diagram showing key regulators of HIF1α in hypoxia and normoxia. During normoxia HIF1α is constantly hydroxylated by prolyl hydroxylases (PHDs), that use oxygen (O2) and iron (Fe2+), thus leading to subsequent ubiquitination and proteasomal degradation of HIF1α. In contrast, under hypoxia or due to hypoxia mimicking factors (such as Roxadustat treatment), PHDs are inhibited, thus enabling the HIF1α subunit to bind to the HIF1β subunit. This HIF-complex can migrate to the nucleus, bind a hypoxia responsive element (HRE), and lead to the activation of several genes involved in angiogenesis, glycolysis, proliferation, and survival [42]. **C** Caspase 3/7 staining of chips in hypoxia was compared to chips in normoxia to show respective areas of apoptosis. **D** Quantification of the Caspase 3/7 assay staining depicting mono-, and co-cultured cells in normoxia and hypoxia when treated with Gemcitabine or Gemcitabine with Roxadustat. The data was compared with Ordinary one-way ANOVA and Tukey’s multiple comparison test and shown are mean and SD (****p ≤ 0.0001, ***P ≤ 0.001, **P ≤ 0.01, *P ≤ 0.05), (*n* = 3, *N* = 2). **E** Reactive Oxygen Species (ROS) staining with ROS marker, Hoechst for nuclei staining and Propidium Iodide (PI) for dead cell staining in PDAC monoculture. **F** Quantification of ROS intensity to the total cell count in hypoxia samples was normalized to normoxia samples comparing untreated samples with samples treated with Gemcitabine with or without Roxadustat. **G** Monocultures treated with 500 nM Gemcitabine (G), 1 uM Roxadustat (R), 1 nM Echinomycin (E) and 10 uM Kc7f2 (K). **H** Co-cultures treated with 500 nM Gemcitabine (G), 1 uM Roxadustat (R), 1 nM Echinomycin (E) and 10 uM Kc7f2 (K) to determine the effect of HIF-inhibitors on PDAC treatment. Shown are mean and SD (****p ≤ 0.0001, ***P ≤ 0.001, **P ≤ 0.01, *P ≤ 0.05), (*N* = 3, *n* = 3). The data was analyzed with two-way ANOVA and Sidak’s multiple comparison test. Shown are maximum projections (10× magnification) of PDAC monoculture for all images, imaged on the ImageXpress Micro Confocal (Molecular Devices, US). Scale Bar = 200 um.

Roxadustat (prolyl-hydroxylases blocker) was used as a molecular tool to further understand the influence of HIF-driven signaling in our model (Fig. 5A). In combination with Gemcitabine, Roxadustat increases survival or prevents the effect of Gemcitabine. To understand if the lowering of HIF signaling would result in the enhancement of the effect, HIF-inhibitors were tested: Echinomycin (blocks the binding of HIF-1α to target genes); and Kc7f2 (inhibits the activation of HIF-target genes and suppresses HIF-1α protein accumulation) (Fig. 5G, H) [19, 20]. Kc7f2 does not seem to have an influence on treatment in the concentration tested. Echinomycin, however, has significantly influenced responses to Gemcitabine and prevented the protective effect of Roxadustat. Response to these modulators is generally higher in hypoxia (survival is lower), but the same trend is present in monoculture and co-culture in hypoxia versus normoxia. This highlights the importance of hypoxia/pseudohypoxia driven signaling in PDAC cell survival as well as HIF as a potential vulnerability for targeting of PDAC.

Apoptosis in response to Gemcitabine or Gemcitabine in combination with Roxadustat was confirmed with Caspase 3/7 staining (Fig. 5C). Quantification (Fig. 5D) showed apoptosis induction in response to Gemcitabine, this was decreased in combination with Roxadustat. Higher survival was also observed in untreated samples under hypoxic conditions. ROS formation was also evaluated, ROS accumulation can either be induced via the NADPH oxidase or a reduced rate of the tricarboxylic acid cycle (TCA) in presence of HIF activation [21]. These processes are dictated by the different signalling pathways of different cell types [22]. Surprisingly, PDAC organoids secrete less ROS when cultivated in hypoxia (Fig. 5E, F). Whereas when grown in co-culture with PSCs, ROS content increases, suggesting that the two cell types cope differently with ROS accumulation. Gemcitabine increases ROS content in both monoculture and co-culture. Roxadustat does not seem to have a major influence on the ROS content when compared to untreated samples in normoxia. However, it slightly prevents ROS accumulation only in monoculture under hypoxic conditions.

## Discussion

We here report the development of 3D (co-)culture pancreatic cancer model set ups to study the impact of hypoxia on pancreatic cancer drug response. The PDAC-on-a-Chip model set ups consist of PDAC organoids with or without PSCs, co-cultured in a microfluidic platform under hypoxic or normoxic conditions. Phenotypic and genotypic differences of cells subjected to hypoxia compared to normally used culture conditions were evaluated.

Recently, Organ-on-a-Chip systems have gained increasing attention as these allow for the inclusion of different cell types grown in a 3D environment under flow conditions [23]. In addition, we mimicked hypoxia, which is a relevant player in the PDAC microenvironment. Expression of PDAC specific keratin CK19 was confirmed in combination with αSMA positive PSCs; mono-and co-cultures were subject to drug response studies under hypoxia and normoxia. These model setups were characterized for cell survival and induction of hypoxia. Transcriptional changes were also assessed by qPCR and RNA sequencing. Hypoxia induces KRAS [24], hedgehog signaling associated genes [10], TGF- β expression [25] and p53 accumulation [26]. RNA sequencing also indicates that EGFR was downregulated and MEK driven signaling was upregulated in monoculture under hypoxia. Vimentin upregulation can be explained due to the switch into a more extensive filamentous network [27]. While a decrease in E-cadherin expression is expected due to EMT, an increase of E-cadherin was observed in PDAC organoids. We conclude that this increase comes from the complex function of E-cadherin upon retaining an epithelial phenotype in cancer for regulating tumorigenicity [28]. Moreover, a loss of LGR5 was observed, which was demonstrated by Emery [29], who showed the ability of tumor cells switching between expressing LGR5 in normoxia and repression in hypoxic conditions. As expected, glycolysis was upregulated, which is associated with favoring HIF-1α signaling and a shift towards lactate production [30]. EMT related genes seemed to be highly upregulated in mono and co-cultures in hypoxia, as well as genes involved in the p53 pathway. DNA repair and oxidative phosphorylation pathways were upregulated in normoxia.

After characterizing our model setups and response to culture conditions, the aim was to understand how changes observed in hypoxic conditions could contribute to the rise of molecular vulnerabilities. Gemcitabine was combined with compounds such as SN38 (topoisomerase inhibitor), Metformin (GDP2 inhibitor), Syrosingopine (MCT inhibitor), Erlotinib (EGFR inhibitor), Trametinib and PD0325901 (MEK inhibitors) and MK2206 (AKT inhibitor) (Table S3).

PDAC mono- and co-cultures showed limited response to Erlotinib, this is likely a result of EGFR downregulation in response to hypoxia. (Figs. 3 and 4). Syrosingopine, a MCT1 (SLC16A1) inhibitor was also tested, which leads to lactate accumulation, ATP depletion and consequent cell death [31]. The lack of response to Syrosingopine as well as to Metformin seems to be a result of the downregulation and less dependence to their targets under hypoxic conditions [32, 33]. These compounds represent group A (Fig. 3), where cells were less responsive in co-culture.

Interestingly, Group B (Fig. 3), induces a reduced survival in co-culture compared to monoculture, these molecules seem to target PSCs as well, such as SN38 (topoisomerase inhibitor). In addition, we found that Trametinib, in the concentration tested, affected monocultures less than the co-cultures (~80% PSCs), suggesting a relevant role of MAPK signaling also in the PDAC stroma. This was confirmed by the response of the co-culture in hypoxia to PD0325901 (MEK inhibitor). The PI3K/Akt pathway has been long considered a relevant target in PDAC [34]. Akt inhibitor MK-2206 decreased cancer cell viability and increased efficacy of Gemcitabine in co-cultures, in both hypoxia and normoxia. Although the stroma plays a role in chemotherapy resistance, it was also shown that elimination of stromal cells led to tumor progression towards a more aggressive phenotype, concluding that the involvement of stroma in PDAC is highly complex and needs to be better understood and effectively targeted [35–38].

In order to understand the role of hypoxia related signaling in normoxic conditions, Roxadustat, HIF-prolyl hydroxylase (PHD) inhibitor, was combined with Gemcitabine [39]. Roxadustat improved survival of mono- and co-cultures, in normoxia and hypoxia indicating a role of HIF driven signaling in normoxia, that becomes even more important in hypoxia. Despite HIF-1α expression in monoculture, responses to Roxadustat suggest that stabilization of HIF-1α in normoxia, and in particular hypoxia contributes to limited response to Gemcitabine, indicating a relevant role of PHD regulation and consequent HIF-1α signaling in PDAC. Additional ROS accumulation studies showed, that ROS accumulation is decreased in hypoxia in monoculture and increased in co-culture compared to normoxia. Also, when cells were treated with Roxadustat under hypoxic conditions, the ROS content decreased, while it increased under the treatment with Gemcitabine alone. The produced ROS likely contributes to HIF stabilization in hypoxia, and to accumulation of HIF under normoxic conditions in co-culture [40]. This is supported by the RNA Seq analyses. Other studies have shown that HIF activation maintains low ROS levels in response to suppression of the TCA cycle during hypoxia in some cell types, which is what we observe in PDAC organoid monocultures [41].

Together these findings provide evidence that hypoxia initiates specific molecular programs in PDAC organoids in mono- and co-cultures that impact response to different classes of compounds. These results also suggest that targeting hypoxia driven signaling could lead to the effective targeting of tumor cells and potentially improve response to conventional and targeted therapies.

## Materials and methods

The materials and methods are listed and described in the Supplementary Materials and Methods.

## Supplementary information

Supplementary Information

Supplementary Figure S1

Supplementary Figure S2

## Data Availability

The data can be found on GEO: under the accession number GSE222482. The code used to generate the results and figures for RNA Sequencing can be found here: https://github.com/DSchreyer/geyer-hypoxia.
