# Supplementary material for: A microfluidic-based PDAC organoid system reveals the impact of hypoxia in response to treatment
Source: Cell Death Discov. 2023 Jan 21;9:20. doi: 10.1038/s41420-023-01334-z (PMC9867742; doi:10.1038/s41420-023-01334-z)
Supplement: Supplementary file 1 — Supplementary Information [file 41420_2023_1334_MOESM1_ESM.docx]

**Supplementary Information**

**Supplementary Materials and Methods**

**Cell culture**

PDAC organoids were acquired from resected tumours from patient 65 (The 'Ethikkommission der FAU' agreed to the study protocol with the number 167_16 Bc and informed patient content was obtained). The organoids were harvested with fine-needle biopsies and the final organoid material was received from Friedrich-Alexander-University Erlangen-Nuremberg, where they were authenticated via STR analysis and compared to donor germline DNA. The organoids were cultured in a 6-well plate in 10 μl growth factor reduced (GFR-) Matrigel (Corning®, 356231) droplets. After seeding, the plate was placed in the incubator with bottom-side up for 15 min. The organoids were cultured in hCPLT-1 medium (**Table S1**), produced according to the SOP Pancreatic Tumor Organoids. The cells were harvested using Cell Recovery Solution (Corning®, 354253) and placed on ice for 30 min, spun down at 200 g for 5 min, resuspended in DMEM+++, counted and used.

***Table S1****: Medium for PDAC organoid culture and Co-culture in the OrganoPlate®*

| **Reagent** | **Supplier** | **Stock Concentration** | **Volume** |
| --- | --- | --- | --- |
| Advanced DMEM/F12 (1% GlutaMax, 1% HEPES, 0,2% Primocin) | Gibco, #12634-028;  Gibco, #35050-061;  Gibco, #15630-080;  Invivogen # ant-pm-2 | NA; 200 mM; 1 M; 50 mg/ml | 36.42ml |
| R-Spondin conditioned media | N.A. | NA | 10ml |
| Wnt3a/Wnt3a-Afm conditioned medium | N.A. | NA | 50ml |
| mNoggin | Peprotech, # 250-38 | 100µg/ml | 100µl |
| B-27 | Gibco,  # 17504-044 | 50X | 2ml |
| N-acetylcysteine | Sigma-Aldrich,  # A9165-5G | 500mM | 250µl |
| Nicotinamide | Sigma-Aldrich,  # N0636 | 1M | 1ml |
| hEGF | Gibco,  # PMG8043 | 500µg/ml | 10µl |
| FGF10 | Peprotech,  # 100-26 | 1mg/ml | 10µl |
| A83-01 | Tocris, # 2939 | 25mM | 2µl |
| Gastrin | Tocris, # 3006 | 100µM | 10µl |
| Primocin | Invivogen,  # ant-pm-2 | 50mg/ml | 200µl |
| Y-27632 Dihydrochloride | Sigma, # Y0503 | 10.5mM | 100µl |
|  |  |  |  |

Pancreatic stellate cells (PSCs, Klon 2.2) were obtained from Marburg University [43] and cultured in DMEM medium (DMEM (Gibco, 10829-018) + 1% Pen/Strep + 1% Glutamax + 1% Hepes). The cells were passaged when they reached 90% confluency. To detach the cells from the flasks, Trypsin 2.5% (ThermoFisher, 15090046) was used. Both cell types were grown in the OrganoPlate® at 37℃ in a humid atmosphere of either 95% O_2_ and 5% CO_2_ or under 1% O_2_, 5% CO_2_ and 94% N_2_.

**Microfluidic OrganoPlate® culture**

The OrganoPlate® 2-lane was used for microfluidic cell cultures. The OrganoPlate® 2-lane consists of 96 microfluidic chips in a 384-wellmicrotiter plate format (Mimetas, cat nr). In the OrganoPlate® 2-lane (Supplementary Figure 1) each chip consists of 2 channels: a gel channel and a perfusion channel. Each chip connects four wells: ECM gel is filled from the gel inlet (A1), and medium is added to the chip from the perfusion inlet (A2) and the perfusion outlet (A4). The observation window (A3) is used to monitor the culture. In the OrganoPlate® 2-lane, GFR-Matrigel (8.3 – 9.4 mg /mL) was used as an ECM. PDAC organoids and/or PSCs were counted and resuspended in GFR-Matrigel. A cell density of 8000 PDAC organoids and 4000 PSCs in 2 μl of GFR-Matrigel were seeded per chip to the gel inlet and incubated for 15 min at 37℃ and 5% CO_2_. After incubation, medium was added to the in- and outlets and the plate was placed in the incubator on a rocking platform at either 1% O_2_ or 20% O_2_ content.

**Immunostaining**

The OrganoPlates® were fixated with 3.7% Formaldehyde (Sigma, cat# 252549-1L) in HBSS (+Ca/Mg) (Sigma, cat# 55037C-1000ML) for 15 min. The plates were washed twice with PBS (Gibco, cat# 70013065), for 5 min each. PBS was added to all chips, the plate was sealed and stored until used for immunostaining. For staining the plates, they were kept on a rocking device (interval: 2min, 1 degree) during all incubation steps. The cells were first washed for 5 min with washing solution containing 4% FCS (Gibco/ATCC, cat# A13450) in PBS, permeabilized for 10 min with permeabilization buffer containing 0.3% Triton X-100 (Sigma, cat#T8787) in PBS and washed again for 5 min. The cells were then blocked with 2% FCS, 2% BSA (Sigma, cat# A2153), 0.1% Tween20 (Sigma, cat# P9616) in PBS for 45 min. The primary antibody (**Table S2**) was prepared in blocking solution and added to the plate for 24h at RT. The secondary antibody (**Table S2**) was prepared in blocking solution and added to the plate after washing the plate twice for 3 min. The second antibody was incubated for 24 h and the plate was washed again twice for 3 min each. The cells were then washed with PBS once for 1 minute and the plate was filled with PBS, sealed and kept in the fridge until imaging.

***Table S2:*** *Antibodies used for immunofluorescent staining*

| **Antibody** | **Supplier, Cat. No** | **Dilution** |
| --- | --- | --- |
| **Primary Antibodies** | | |
| CK19 (mouse) | Thermo Fisher, #MA5-12663 | 1:100 |
| Vimentin (rabbit) | Abcam, #ab92547 | 1:100 |
| aSMA (mouse) | Sigma-Aldrich, #A2547 | 1:100 |
| **Secondary Antibodies** | | |
| Donkey-anti-mouse 647 | Life Technologies, #A21449 | 1:250 |
| Goat-anti-rabbit 488 | Life Technologies, #A32731 | 1:250 |
| Goat-anti-mouse 555 | Life Technologies, #A32727 | 1:250 |

**Chemotherapeutics**

Chemotherapeutics testing was performed 4 days after the PDAC organoids (and PSCs) had been seeded. The cells were exposed to 1uM of the chemotherapeutics (**Table S3**). 0.1% DMSO was used as a vehicle control and no cell death was observed.

***Table S3:*** *Compounds used*

| **Reagent** | **Supplier** | **Function** |
| --- | --- | --- |
| Erlotinib | Selleck Chemicals, # S1023 | EGFR inhibitor |
| Mirdametinib (PD0325901) | Stemcell Technologies  #72184 | MEK1/2 inhibitor |
| SN38 | Selleckhem, #S4908 | DNA topoisomerase I blockage |
| MK2206 | Selleck Chemicals, #S1078 | AKT1/2/3 inhibitor |
| Trametinib | Selleck Chemicals, #S2673 | MEK1/2 inhibitor |
| Metformin HCL | Selleck Chemicals, #S1950 | GPD2 inhibitor |
| Syrosingopine | Sigma-Aldrich, #SML1908-5MG | MCT1/MCT4 blocker, Glycolysis inhibitor |
| Gemcitabine | Selleck Chemicals, #S1149 | Blocks DNA synthesis |
| Roxadustat | Selleck Chemicals, #S1007 | HIF-PHD inhibitor |
| Echinomycin | Sigma-Aldrich, #SML0477 | blocks the binding of HIF-1α to target genes |
| KC7F2 | Sigma-Alrich, #SML1043 | suppresses HIF-1α protein accumulation |

**CellTiter-Glo® 3D Viability Assay**

The CellTiter-Glo® 3D Cell reagent (Promega, G9681) was thawed and equilibrated at RT for 30 min. The reagent was mixed 1:1 with HBSS and added to the plate, 25 μl was added to both the in- and outlet of the perfusion channels. After a 30 min incubation on the OrganoFlow® (8 min interval, 14º inclination) at 37℃, the luminescence was measured on the Fluororeader (Fluoroskan Ascent FL).

**Caspase-3/7 Green Apoptosis Assay**

To determine which cells undergo apoptosis in the co-culture, the Incucyte® Caspase-3/7 Green Apoptosis Assay (Incucyte, 4440) was performed. The Caspase-3/7 Green Apoptosis Assay Reagent was diluted 1:1000 and 30 μl solution was added to the perfusion inlet and outlet (60 μl/chip). The reagent was incubated on the rocker for 24 hours and then imaged using the ImageXpress Micro XLS confocal microscope. Images were analysed using Fiji (Version 2).

**Live and Dead Assay**

Calcein-AM (Lifetechnologies, C3099, 1:2000), Hoechst 3342 (1:2000 dilution) and DraQ7 (BioStatus, DR71000, 1:1000) were used for staining live and dead cells and the nucleus. The reagents were added to medium and 25 μl were distributed to each the perfusion inlets and outlets. The mix was incubated for 30 min and fluorescent imaging was performed.

**Hypoxia Image-IT assay**

The Image-IT probe was prepared in a final concentration of 5 µM in media. As a positive control glucose oxidase 2 enzyme system with 2 U/ml glucose oxidase 2, 1 mM glucose and 120 U/ml Catalase was used. The medium in the chips was replaced with the Image-IT probe and the cells were imaged after 24 hours.

**ROS assay**

Cell culture medium was removed from all cells, and these were washed with HBSS (Sigma H6648-500ML) buffer for 3 min. DHR123 (Dihydrorhodamine 123, ≥95% Sigma-Aldrich D1054-2MG) was diluted in a 1:1160 ratio in medium and it was then added to the inlet and outlets of the OrganoPlate®. The mix was incubated for 15 min at 37°C, 5% CO2 and 90% humidity, on the OrganoFlow, under standard perfusion settings. Chips were washed 2x 3 min with HBSS and nuclear staining Hoechst was added (Hoechst 33342, Trihydrochloride, Trihydrate – 10 mg/mL solution in water, ThermoFisher H3570) in a 1:2000 dilution, in addition to a propidium iodide solution in a 1:200 dilution to the inlets and outlets for 20 min. The plate was then imaged with a confocal microscope Micro XLS-C High Content Imaging Systems (Molecular Devices, US).

**RNA isolation, cDNA synthesis and qPCR**

To determine the gene expression of the PDAC organoids, RNA was isolated from the cells, transformed into complementary DNA (cDNA) and analysed with quantitative Real-Time PCR (qPCR).  RNA was extracted using the RNeasy® MicroKit (Qiagen, 74004) following manufacturer’s protocol. Next, cDNA was synthesized from the isolated RNA. A master mix consisting of random primers (Invitrogen, 48190011), dNTPs (Invitrogen, 10297018) and UltraPure™ DNase/RNase-Free Distilled Water (ThermoFisher, 10977035) was distributed into tubes containing the RNA samples and the tubes were spun down. The samples were put in the thermocycle (LightCycler® 96 Instrument, Roche, 0515916001) and heated to 65°C for 5 min to ensure that the RNA had denatured. After heating, a new master mix was added to the samples containing M-MLV (reverse-transcriptase) (Invitrogen, 28025013), RNase OUT (40 U/µl) (Invitrogen, 10777019), 0.1 M DTT (Invitrogen, 28025013) and 5x first strand buffer (Invitrogen, 28025013). The samples were first incubated at RT to allow for primer extension and then transferred to the thermocycler. A program was started that first, heated up to 37 °C for 50 min which allowed cDNA polymerization, and second heated up to 70°C for 15 min for enzyme inactivation. Finally, a PCR-mix was added to the cDNA samples consisting of a specifically designed primer mix. First Start Essential DNA Green Master and RNase free water (Roche, 064027112001). The samples were pipetted into the LightCycler® 480 Multiwellplate 96 (Roche, 0472969200) and the plate was sealed with the LightCycler® Sealing foil to prevent evaporation. The plate was spun down and put into the LightCycler® 480 Real-Time PCR system (Roche). Data was analysed using the LightCycler® 96 software and Microsoft Excel®.

**Bulk RNA Sequencing**

Mono- and co-culture samples grown in normoxia and hypoxia were sent out for bulk RNA sequencing and data analysis to Single Cell Discoveries. Normalization was done using DESeq2 rlog method [44]. The fold change as well as the log2 fold change were calculated for each comparison for differential gene expression:

*log2FC = log2NormCounts1 - log2NormCounts*

*FC = 2log2FC*

A gene set enrichment analysis was carried out based on ranking the genes according to the FC calculated using the R-package fgsea and its function ‘fsgeaMultilevel’[45]. Here, the HALLMARK database was used. A positive NES and a p.adjusted value <0.15 means upregulated in normoxia and a negative NES and a p.adjusted value <0.15 means upregulated in hypoxia.

**Statistical analyses**

All statistical analyses were conducted in GraphPad Prism version 9 (GraphPad Software, San Diego, CA, USA) and data was presented as mean ± standard deviation (SD). Differences in survival were assessed using one-way or two-way ANOVA in combination with respective Tukey’s multiple comparison test or Sidak`s multiple comparison. A statistical significance of p ≤ 0.05 was maintained. Independent experiments are denoted by N, while replicates per experiment are denoted by n. As organoids generally show a higher variability, we excluded outliers, where survival was over 20% higher or lower compared to the mean. Sample size was chosen based on the variation and standard deviation between samples to ensure significance of the data. F-tests, descriptive statistics and row analysis were performed to ensure similar variance between the groups.

**Supplementary Figures and Tables**

**Figure legends**

**Figure S1:** **PDAC organoid and PSC grown in the 2-lane OrganoPlate®**. The PDAC organoids were grown for 7 days in co-cultivation with PSCs. PDAC organoids appear as round cells with a big lumen, whereas PSCs grown in DMEM on a plastic surface appeared flattened, myofibroblast-like. However, when grown in Matrigel and in media containing N-acetylcysteine, their phenotype changed resulting in senescence and significantly slower growth. In addition, these show decreased Collagen I, CTGF and TGF- β expression and, the cells appear elongated forming a filamentous network (Jesnowski et al., 2005). **A.** 4x Phase-contrast images of OrganoPlate® cultures of pancreatic ductal adenocarcinoma organoids in monoculture or co-culture with PSCs in normoxia or hypoxia. Scale bar: 200 µm. **B.** Immunofluorescent staining of PDAC organoids after 7 cultivation days with CK19 (red) and of PSCs with Vimentin (red) and ɑ-SMA (red). Nuclear staining with NucBlue. 10x magnification, Scale bar: 200 µm. Images acquired on the ImageXpress Micro Confocal (Molecular Devices). Fluorescent images are shown in maximum intensity projections.

***Figure S2.:*** ***Top up- and downregulated pathways in mono- and co-cultures. A.*** *pathways up-and downregulated in co-culture. In* ***B.*** *monocultures are shown. Pathways upregulated in hypoxia are below 0 and pathways upregulated in normoxia are depicted above 0*

**Table S4**: RNA Seq analysis raw data. Fc indicates the foldchange, whereas l2fc indicated the log of the fold change

| **Mono_Normoxia** | **Mono_Hypoxia** | **l2fc** | **fc** | **Gene** |
| --- | --- | --- | --- | --- |
| 9.935262 | 9.92453 | 0.010731 | 1.007466 | AKT1 |
| 9.319402 | 9.165072 | 0.15433 | 1.112904 | AKT2 |
| 3.688348 | 3.710177 | -0.02183 | 0.984983 | AKT3 |
| 8.118332 | 8.078857 | 0.039475 | 1.02774 | EGFR |
| 8.686385154 | 8.853786455 | -0.1674 | 0.890445 | MEK1 |
| 9.282092548 | 9.523923504 | -0.24183 | 0.845671 | MEK2 |
| 8.729693 | 8.6917 | 0.037993 | 1.026685 | SLC16A1 |
| 8.055086 | 8.009076 | 0.04601 | 1.032405 | GPD2 |
| 7.48858 | 7.519882 | -0.0313 | 0.978537 | HIF1A |
|  |  |  |  |  |
| **Co_Normoxia** | **Co_Hypoxia** | **l2fc** | **fc** | **Gene** |
| 9.585415663 | 9.758927898 | -0.17351 | 0.886681 | AKT1 |
| 8.870700852 | 9.201562629 | -0.33086 | 0.795061 | AKT2 |
| 6.422478 | 6.079282 | 0.343196 | 1.268564 | AKT3 |
| 8.296255 | 8.116367 | 0.179888 | 1.132796 | EGFR |
| 9.095994 | 9.161025 | -0.06503 | 0.955925 | MEK1 |
| 9.10317421 | 9.554683984 | -0.45151 | 0.731277 | MEK2 |
| 8.907944 | 8.693931 | 0.214013 | 1.15991 | SLC16A1 |
| 7.959956 | 7.485948 | 0.474008 | 1.388962 | GPD2 |
| 7.143703 | 6.62905 | 0.514652 | 1.42865 | HIF1A |
|  |  |  |  |  |
| **Mono_Normoxia** | **Co_Normoxia** | **l2fc** | **fc** | **Gene** |
| 9.935261508 | 9.585415663 | 0.349846 | 1.274424 | AKT1 |
| 9.319401622 | 8.870700852 | 0.448701 | 1.364811 | AKT2 |
| 3.68834775 | 6.422478154 | -2.73413 | 0.150295 | AKT3 |
| 9.050540754 | 9.967149824 | -0.91661 | 0.529753 | MEK1 |
| 7.4885796786 | 7.143702510 | 0.34487 | 1.2700428 | HIF1A |
|  |  |  |  |  |
| **Mono_Hypoxia** | **Co_Hypoxia** | **l2fc** | **fc** | **Gene** |
| 9.924530342 | 9.758927898 | 0.165602 | 1.121634 | AKT1 |
| 9.165072094 | 9.201562629 | -0.03649 | 0.975024 | AKT2 |
| 3.710176669 | 6.079281975 | -2.36911 | 0.193566 | AKT3 |
| 9.108322965 | 9.53222328 | -0.4239 | 0.745407 | MEK1 |
| 7.5198818922 | 6.62905024 | 0.890831 | 1.8542446 | HIF1A |
