# Supplementary figures and images for: A microfluidic-based PDAC organoid system reveals the impact of hypoxia in response to treatment
Source: Cell Death Discov. 2023 Jan 21;9:20. doi: 10.1038/s41420-023-01334-z (PMC9867742; doi:10.1038/s41420-023-01334-z)

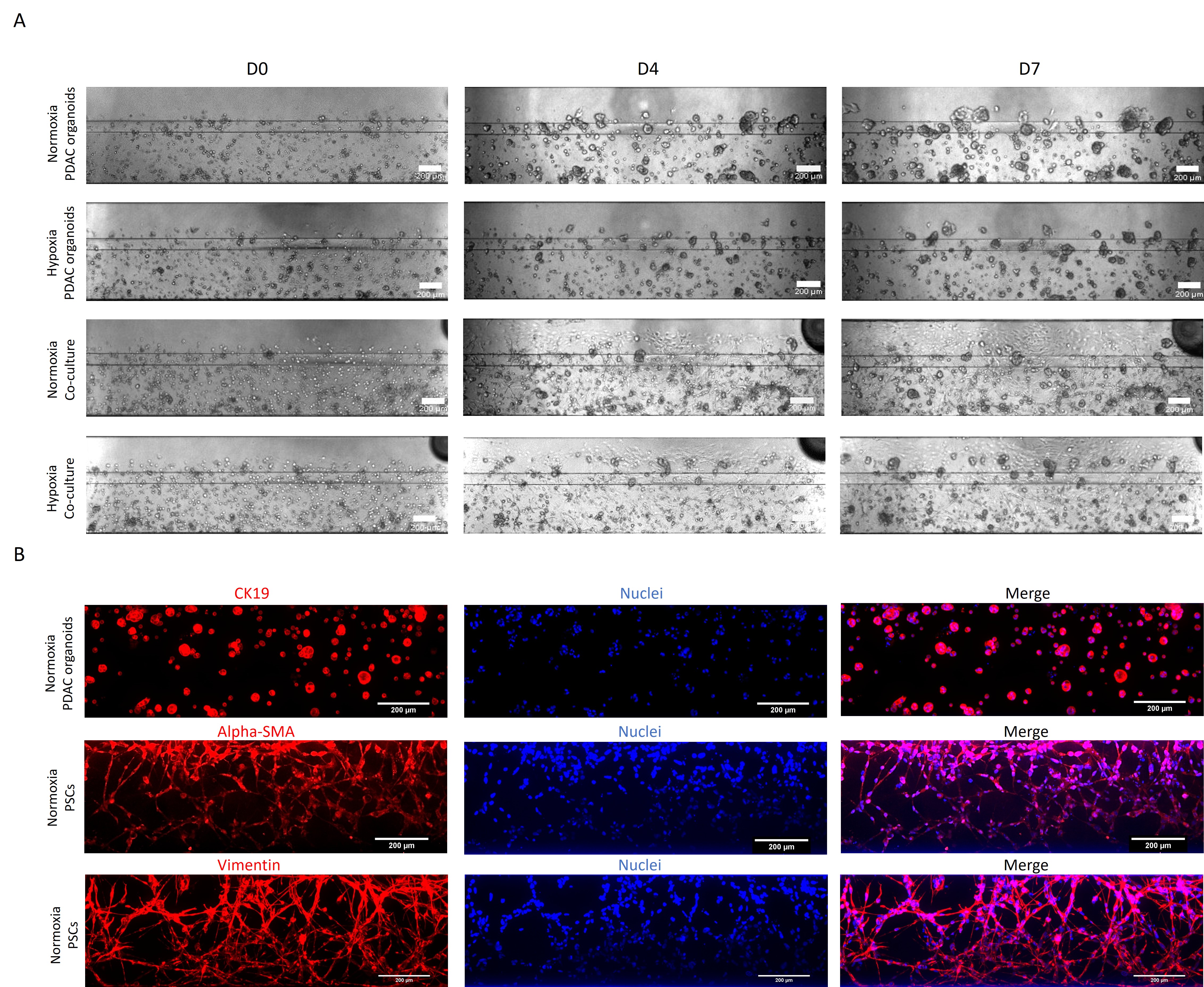

Supplement: Supplementary file 2 — Supplementary Figure S1 [file 41420_2023_1334_MOESM2_ESM.jpg]

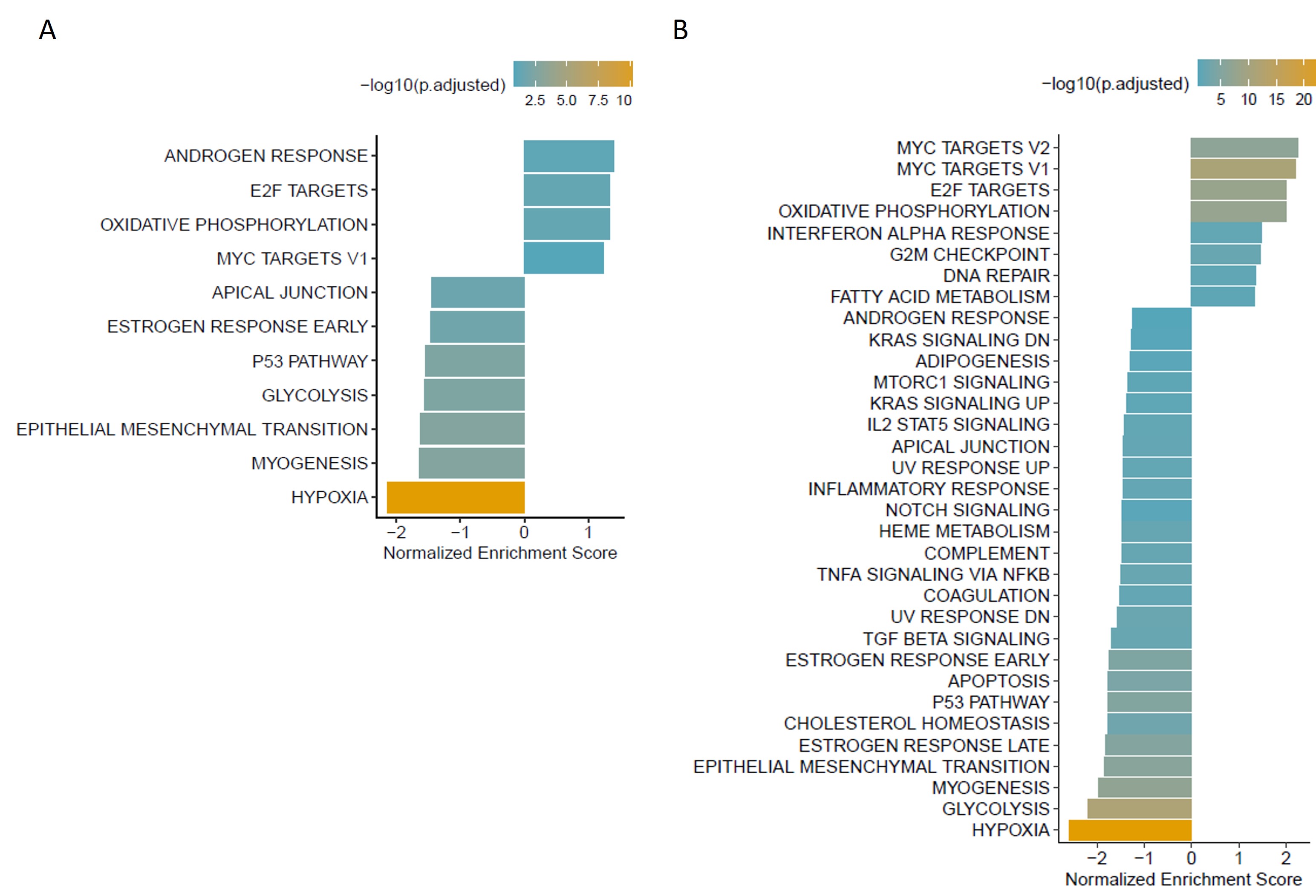

Supplement: Supplementary file 3 — Supplementary Figure S2 [file 41420_2023_1334_MOESM3_ESM.jpg]
